# Fixing the Achilles Heel of Pfizer’s Paxlovid for COVID-19 Treatment

**DOI:** 10.1021/acs.jmedchem.4c01342

**Published:** 2024-07-05

**Authors:** Lennart Brewitz, Christopher J. Schofield

**Affiliations:** † Chemistry Research Laboratory, Department of Chemistry and the Ineos Oxford Institute for Antimicrobial Research, University of Oxford, 12 Mansfield Road, OX1 3TA Oxford, United Kingdom

## Abstract

Nirmatrelvir (PF-07321332), a first-in-class inhibitor of the severe acute respiratory syndrome coronavirus-2 (SARS-CoV-2) main protease (M^pro^), was developed by Pfizer under intense pressure during the pandemic to treat COVID-19. A weakness of nirmatrelvir is its limited metabolic stability, which led to the development of a combination therapy (paxlovid), involving coadministration of nirmatrelvir with the cytochrome P450 inhibitor ritonavir. However, limitations in tolerability of the ritonavir component reduce the scope of paxlovid. In response to these limitations, researchers at Pfizer have now developed the second-generation M^pro^ inhibitor PF-07817883 (ibuzatrelvir). Structurally related to nirmatrelvir, including with the presence of a trifluoromethyl group, albeit located differently, ibuzatrelvir manifests enhanced oral bioavailability, so it does not require coadministration with ritonavir. The development of ibuzatrelvir is an important milestone, because it is expected to enhance the treatment of COVID-19 without the drawbacks associated with ritonavir. Given the success of paxlovid in treating COVID-19, it is likely that ibuzatrelvir will be granted approval as an improved drug for treatment of COVID-19 infections, so complementing vaccination efforts and improving pandemic preparedness. The development of nirmatrelvir and ibuzatrelvir dramatically highlights the power of appropriately resourced modern medicinal chemistry to very rapidly enable the development of breakthrough medicines. Consideration of how analogous approaches can be used to develop similarly breakthrough medicines for infectious diseases such as tuberculosis and malaria is worthwhile.

Nirmatrelvir (PF-07321332; **1**) is a first-in-class small-molecule inhibitor of the severe acute respiratory syndrome coronavirus-2 (SARS-CoV-2) main protease (M^pro^/3C-like protease), which is used clinically in combination with ritonavir under the brand-name paxlovid to treat and/or hinder SARS-CoV-2 infections (Figure 1a).^1,2^ M^pro^ is a nucleophilic cysteine protease that catalyzes the hydrolysis of viral polyproteins pp1a/1ab to give functional nonstructural proteins;^3−5^ M^pro^ inhibition disrupts the viral life cycle and halts viral replication.^6−8^ Work leading to the development of paxlovid, which was carried out under intense time and social pressure during the COVID-19 pandemic, was a major breakthrough, because it validated inhibition of M^pro^ as a treatment for COVID-19.^1,2^ Paxlovid treatment complements vaccination campaigns and provides a safe means to cure infections in vulnerable groups;^2,9^ along with several other clinically approved M^pro^ inhibitors subsequently reported by others,^10−14^ it has contributed to reducing the death rate associated with SARS-CoV-2 infections and helped enable a return to pre-COVID-19 lifestyles.

**Figure 1:**
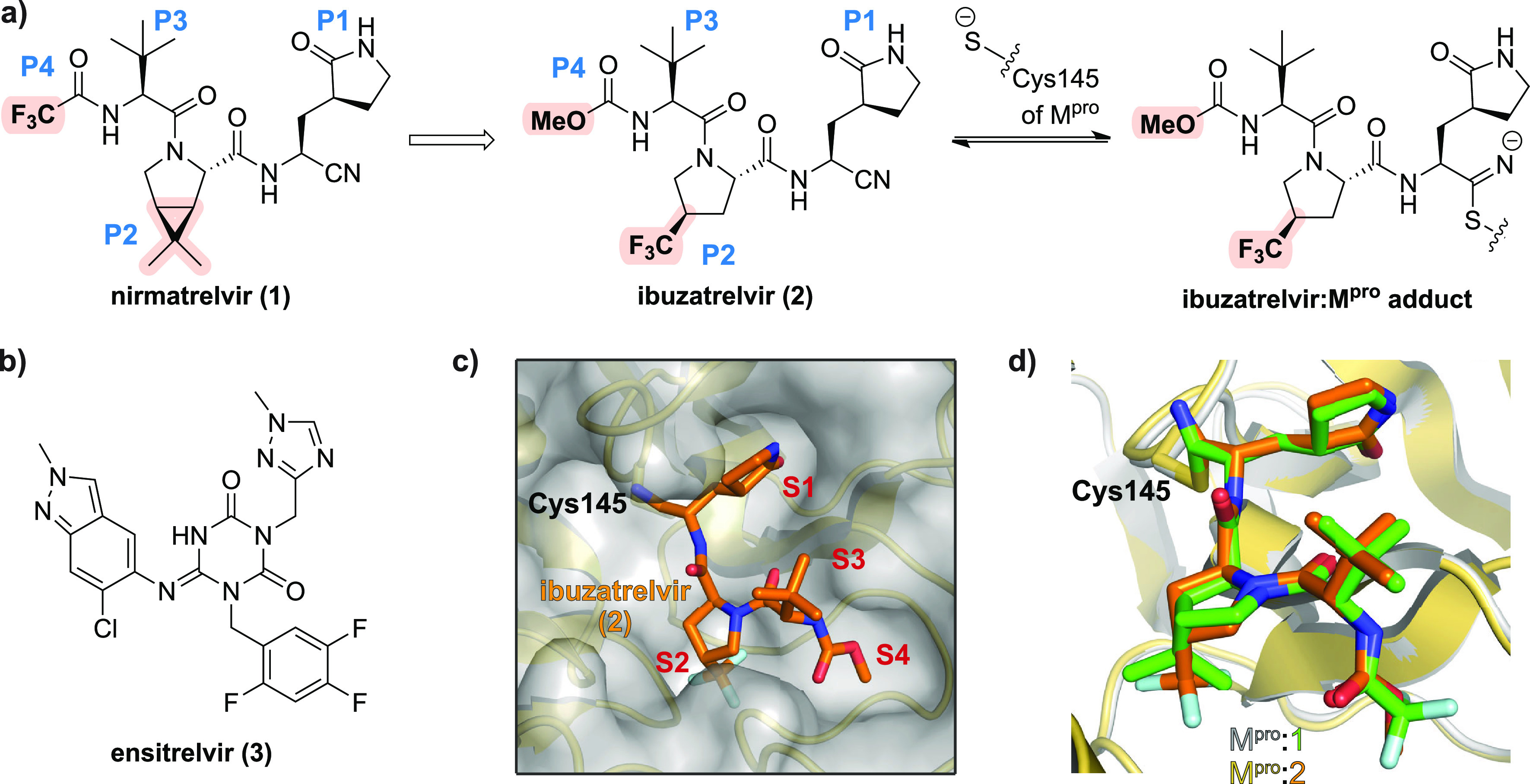
Like nirmatrelvir, ibuzatrelvir inhibits SARS-CoV-2 M^pro^ via reversible covalent reaction. (a) Ibuzatrelvir (PF-07817883; **2**)^15^ is structurally related to nirmatrelvir (PF-07321332; **1**)^1^ and inhibits SARS-CoV-2 M^pro^ via reversible covalent reaction with the nucleophilic thiolate of Cys145 (deprotonated by His41). (b) Ensitrelvir (S-217622; **3**) inhibits M^pro^ by tight binding to the active site via noncovalent interactions.^10^ (c) View from a crystal structure of SARS-CoV-2 M^pro^ (gold cartoon and gray surface) in complex with ibuzatrelvir (orange carbon backbone; PDB ID: 8V4U(15)); the nitrile-derived thioimidate is located in an oxyanion hole (formed by the main chain amide NHs of Cys145 and Gly143). (d) Ibuzatrelvir (orange carbon backbone; PDB ID: 8V4U(15)) and nirmatrelvir (green carbon backbone; PDB ID: 7TE0(19)) adopt similar conformations when bound to SARS-CoV-2 M^pro^.^15^ P1-P4-equivalent positions are in blue; S1-S4 subsites are in red.

Recently, researchers from Pfizer have reported on the development of PF-07817883 (ibuzatrelvir; **2**) as a nirmatrelvir-derived second-generation orally active M^pro^ inhibitor and clinical candidate to treat SARS-CoV-2 infections (Figure 1a).^15^ The development of **2** addresses a major limitation of **1**, which compromises its unrestricted use in all patient groups, *i*.*e*., its limited metabolic stability requiring twice daily coadministration with ritonavir (taken as separate tablets), an inhibitor of the cytochrome P450 oxygenase CYP3A4 that metabolizes **1**,^1,16^ to boost its activity.^1,2^ The use of the ritonavir ingredient of paxlovid limits the clinical scope of paxlovid, because ritonavir has side effects and its use affects the efficacy of other medications that are commonly prescribed in vulnerable groups (and *vice versa*).^15,17^

Nirmatrelvir (**1**) was carefully optimized during initial structure–activity relationship studies, which revealed that the trifluoroacetate group at the P4-equivalent position was particularly important in increasing potency in infected cells and metabolic stability.^1^ The structure of **2** is similar to that of **1** with the exceptions that it bears a methylcarbamate at the P4-equivalent position and, strikingly, that a (4*R*)-4-trifluoromethylproline group substitutes the bicyclic (1*R*,5*S*)-3-azabicyclo[3.1.0]hexane proline derivative of **1** at the P2-equivalent position (Figure 1a). Both **1** and **2** employ a nitrile group for reversible covalent reaction with the nucleophilic cysteine residue of M^pro^, *i.e*., Cys145, to give a thioimidate analogue of the acyl-enzyme complex formed during the M^pro^-catalyzed hydrolysis of the viral polyproteins pp1a/1ab (Figure 1);^1,15^ the nitrile group of **1** can be substituted for an isoelectronic alkyne, resulting in apparently irreversible covalent reaction with Cys145.^18^ Importantly, crystallographic analyses reveal that both **1** and **2** adopt a similar conformation when bound to M^pro^ (Figure 1d).^1,15^ However, it should be noted that efficient M^pro^ inhibition can also be achieved by small molecules which bind to the M^pro^ active site via noncovalent interactions, as is the case for the clinically used drug ensitrelvir (Figure 1b).^10^

The development of PF-07817883 (**2**) by Pfizer highlights the synergistic effects of modifying the groups at the P2- and P4-equivalent positions of **1**, resulting in substantially enhanced metabolic stability in human liver microsomes (HML) and reduced lipophilicity (log *D*) compared to **1** (Table 1). Despite these modifications, **2** maintains dose-dependent high potency *in vitro* and in infected cells (Table 1).^15^ The use of a conformationally more flexible (4*R*)-4-trifluoromethylproline group at the P2-equivalent position of **2** facilitates rotation around the P2/3 amide bond, a property which is proposed to improve the oral absorption of **2** compared to **1**.^15^ The superior oral pharmacokinetics and substantially improved absorption of **2** compared to other derivatives of **1**/**2** tested in animal models, highlight the potential of **2** to outperform **1** in a clinical setting, most importantly because it does not require the presence of ritonavir to hinder its metabolism and so increase activity.^15^ The improvements in pharmacokinetics of **2**, together with its low toxicity/high selectivity and projected few/no detrimental drug–drug interactions, have resulted in its nomination as a clinical candidate.^15^ It is likely that, following thorough clinical testing to demonstrate safety and efficacy in humans, **2** (or a derivative of it) will substantially improve COVID-19 treatment for vulnerable groups.

**Table 1:**
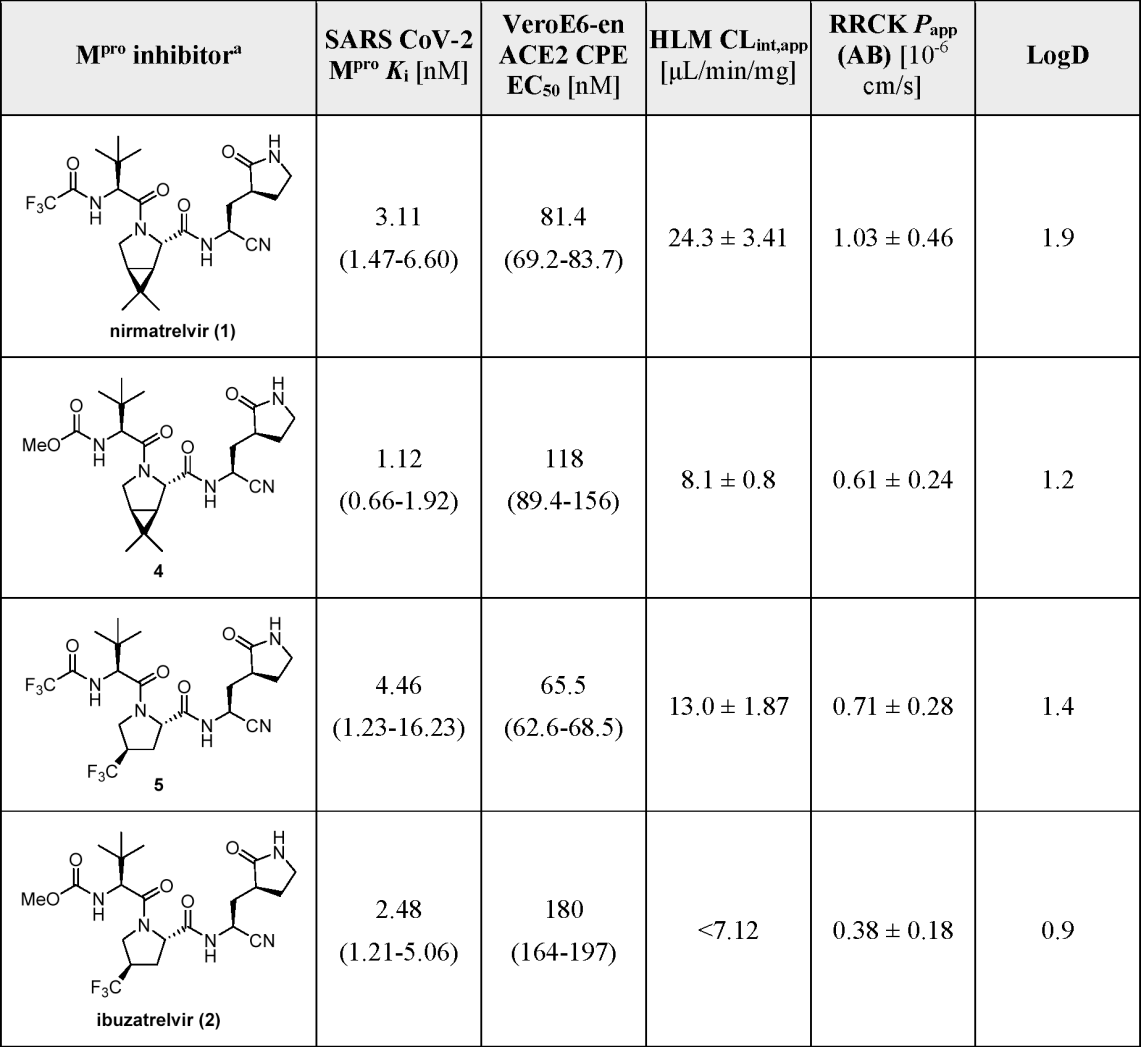
Biochemical and Physicochemical Parameters of Selected Nirmatrelvir (**1**)/Ibuzatrelvir (**2**)-derived M^pro^ Inhibitors^a^^,^^15^

a Abbreviations: *K*_i_, inhibition constant, determined using Förster resonance energy transfer (FRET) assays with isolated recombinant SARS-CoV-2 M^pro^; VeroE6-en ACE2 CPE, inhibition of the SARS-CoV-2-caused cytopathic effect (CPE) in VeroE6 cells enriched for ACE2 receptor expression, determined in the presence of the efflux inhibitor CP-100356; HLM CL_int,app_, total apparent intrinsic clearance (CL_int,app_), determined in NADPH-supplemented human liver microsomes (HLM); RRCK *P*_app_ (AB), absorptive passive permeability measured from apical to basolateral direction, determined in Ralph Russ canine kidney cells (RRCK)-based assays; LogD, lipophilicity, determined at pH 7.4 by the shake-flask method.^15^

Focused work leading to the development of nirmatrevlir (**1**)/paxlovid and, subsequently, **2** was initiated in March 2020 and occurred in a remarkably short timeframe.^20^ It is important to note, however, that these efforts built on extensive pre-existing medicinal chemistry efforts and basic science research, including studies on coronavirus proteases and the pioneering validation of viral proteases as clinically useful targets for human immunodeficiency virus (HIV) and hepatitis C virus (HCV) treatment.^21−24^ The development of **2** (and **1**) is grounded in earlier efforts of scientists at Pfizer to obtain small-molecule antivirals in response to the SARS-CoV-1 outbreak,^25,26^ and pioneering structure–activity relationship studies on M^pro^/3C(-like) protease inhibitors by others.^21^ Among other insights relevant to the development of **1** and **2**, these studies revealed that (i) the nitrile group of substrate-derived inhibitors can covalently react with SARS-CoV-1 M^pro^,^27^ (ii) a γ-lactam group at the P1-equivalent position of substrate-based inhibitors of M^pro^/3C(-like) protease can enhance inhibition potency,^28−30^ and (iii) the bicyclic (1*R*,5*S*)-3-azabicyclo[3.1.0]hexane proline derivative can serve as a leucine isostere.^31,32^

The growing repertoire of (partly) mechanistically distinct M^pro^ inhibitors for COVID-19 treatment highlights the utility of M^pro^ as a viable drug target, something which may in part be attributed to the substrate specificity of M^pro^, which is proposed to differ from that of human proteases. In accord with this proposal, natural substrate-related inhibitors, such as **2**, typically show a relatively low off-target reactivity with human protases (though **2** is reported to also inhibit human cathepsins K/S *in vitro*).^15^ It should be noted that the selectivities of **1** and **2** likely also relate to their high potency and, potentially, also to the reversible nature of their covalent reaction with M^pro^.^1,15^

Other SARS-CoV-2 proteins/protein complexes than M^pro^ are also drug targets, highlighted *inter alia* by the approval of remdesivir and molnupiravir for COVID-19 treatment,^33,34^ though these may not be optimal drugs.^35−38^ The available evidence, however, suggests that M^pro^ may be a particularly attractive long-term drug target, as reflected in its apparent relatively low mutation rate compared to other SARS-CoV-2 proteins such as the spike protein,^39^ and by the observation that **1** and **2** are active against the current major clinically observed SARS-CoV-2 variants.^15,40^ Further, despite the relatively high intrinsic mutation rate of the SARS-CoV-2 genome, the high number of SARS-CoV-2 infections worldwide, and the widespread clinical use of paxlovid, the prevalence of nirmaltrelvir-resistant M^pro^ variants appears relatively low at present.^39,41^

As a first-in-class SARS-CoV-2 M^pro^ inhibitor, nirmatrelvir (**1**) has influenced the development of ibuzatrelvir (**2**),^15^ as well as other substrate-derived M^pro^ inhibitors which are now in clinical use, *i.e*., simnotrelvir (used in combination with ritonavir)^11,12^ and leritrelvir (used without ritonavir).^13,14^ The development of **2**, which has improved properties relative to **1**, illustrates how continued research beyond approval of first-in-class medication can lead to improved drugs. The structure of **2** may be optimized further, *e.g*., with respect to its off-target selectivity, dosing regime, and/or to limit occasional recurrence of symptoms.^42^ Despite exhibiting broad-spectrum pan-coronavirus activity, **2** may be further modified to enhance its potency for the inhibition of coronaviruses other than SARS-CoV-2 for which currently no treatment is available, *e.g*., the Middle East respiratory syndrome-related coronavirus (MERS-CoV); **2** inhibits MERS-CoV M^pro^ with reduced *in vitro* potency compared to SARS-CoV-2 M^pro^.^15^ Hence, continued and appropriately resourced efforts to build on the work of Pfizer and others on M^pro^ inhibitors has the potential to be instrumental in enabling global preparedness for future coronavirus pandemics. In the absence of a short-term pandemic threat, consideration of how such work will be funded is important.^43^

Following prior campaigns to develop HIV and HCV protease inhibitors,^22−24^ the success by Pfizer and others in developing therapeutically useful SARS-CoV-2 M^pro^ inhibitors extends the therapeutic use of protease inhibitors to treat coronavirus infections, indicating that proteases of pathogenic viruses, for which currently no treatment exists, will likely also be viable drug targets. Notably, M^pro^ is a nucleophilic cysteine protease,^3−5^ whereas HIV protease is an aspartyl-protease^44^ and the HCV NS3/4A protease is a serine protease;^45^ hence, different (if not all) mechanistic classes of viral protease are therapeutically tractable.

The M^pro^ work raises the question whether the other nucleophilic cysteine protease encoded by the SARS-CoV-2 genome, *i.e*., the papain-like protease (PL^pro^), is also a good drug target.^46^ Ongoing efforts are targeting PL^pro^; however, unlike M^pro^, PL^pro^ shares a common fold and overlapping substrate scope with certain human nucleophilic cysteine enzymes, *e.g*., deubiquitinases (DUBs),^47,48^ complicating the development of selective PL^pro^ inhibitors. Additionally, early efforts to develop potent inhibitors for PL^pro^ were likely hampered by imperfect assays for isolated recombinant PL^pro^ during the onset of the COVID-19 pandemic.^49,50^ Despite these challenges, pioneering work on substrate-competitive small-molecule SARS-CoV PL^pro^ inhibitors^21,51^ has enabled recent developments of SARS-CoV-2 PL^pro^ inhibitors as lead candidates for drug development,^52−54^ including by researchers from Pfizer.^55^

Overall, Pfizer’s groundbreaking work on the clinical development of nirmatrelvir^1^ (PF-07321332; **1**) and ibuzatrelvir^15^ (PF-07817883; **2**) demonstrates that SARS-CoV-2 M^pro^ and, by implication, main proteases of related coronaviruses are viable clinical targets. From a mechanistic perspective, the demonstration that nitriles are useful electrophiles for the development of clinically useful and safe (substrate-based) inhibitors, which covalently react in a reversible manner with pathogenic cysteine enzymes, is particularly notable and will likely find application in the inhibition of other classes of nucleophilic cysteine enzymes, *e.g*., other proteases and deubiquitinating enzymes. Importantly, the work beautifully illustrates how superficially (to the nonexpert) relatively minor structural modifications to the core of a pioneering inhibitor (**1** and, by implication, other M^pro^ inhibitors) can significantly alter important physicochemical properties and oral bioavailability, leading to an improved medicine (*i.e*., **2**). The work thus highlights a need for continued careful structure–activity relationship studies by medicinal chemists to provide society with both first-in-class and improved treatments for current and future infectious diseases, including in response to resistance. Pioneering work on antibacterials in the 20th century demonstrates the medicinal value of such an approach.^56^
